# A Novel Concept is Needed for Combating Alzheimer’s Disease and NeuroHIV

**DOI:** 10.36959/734/377

**Published:** 2020-08-19

**Authors:** Xiu-Ti Hu

**Affiliations:** Department of Microbial Pathogens and Immunity, Rush University Medical Center, USA

**Keywords:** Alzheimer’s disease, NeuroHIV, Neurotoxicity, Ca^2+^ dysregulation, Medial prefrontal cortex, Pyramidal neurons

## Abstract

Both Alzheimer’s disease (AD) and HIV-associated neurocognitive disorders (HAND) could progress to dementia, a severe consequence of neurodegenerative diseases. Cumulating evidence suggests that the β-amyloid (Aβ) theory, currently thought to be the predominant mechanism underlying AD and AD-related dementia (ADRD), needs re-evaluation, considering all treatments and new drug trials based upon this theory have been unsuccessful. Similar intention for treating HAND, including HIV-associated dementia (HAD), has also failed. Thus, novel theory, hypothesis, and therapeutic strategies are desperately needed for future study and effective treatments of AD/ADRD and HAND. There are numerous potential upstream mechanisms that may cause AD and/or HAND; but it is unrealistic to identify all of them. However, it is realistic and feasible to intervene the downstream mechanism of these two devastating neurodegenerative diseases by blocking the final common path to neurotoxicity mediated by overactivation of NMDA receptors (NMDARs) and voltage-gated calcium channels (VGCCs). Such a combined pharmacological intervention will likely ameliorate neuronal Ca^2+^ homeostasis by diminishing overactivated NMDAR and VGCC-mediated Ca^2+^ dysregulation (i.e., by reducing excessive Ca^2+^ influx and intracellular levels, [Ca^2+^]in)-induced hyperactivity, injury, and death of neurons in the critical brain regions that regulate neurocognition in the context of AD/ADRD or HAND, especially during aging. Here we present a novel theoretical concept, hypothesis, and working model for switching the battlefield from searching-and-fighting the original mechanism that may cause AD or HAND, to abolishing AD- and neuroHIV-induced neurotoxicity mediated by NMDAR and VGCC over activation, which may ultimately improve the therapeutic strategies for treating AD and HAND.

## Introduction

In current treatments and studies focusing on combating against Alzheimer’s disease (AD) and AD-related dementia (ADRD), the reality is that many drug trial investigations, especially conducted during aging, have unfortunately failed [1,2]. The β-amyloid (Aβ) theory, which has been guiding us to fight against AD/ADRD for more than three decades, could unfortunately be wrong. It is likely that we do not really understand the original mechanism that actually causes AD, as well as other devastating neurodegenerative diseases that could also ultimately progress o severe neurocognitive dysfunction, including dementia [3-5]. Under such critical circumstances, we may need to re-consider, plan, explore, and eventually develop a novel theory and hypothesis, which may guide us to establish new therapeutic strategies, and thereby allowing us to continuously combat against AD/ADRD and other related neurodegenerative diseases.

Aging is a significant and growing risk factor for neurodegenerative diseases, including, but not limited, to AD/ADRD [1,2] and HIV-associated neurocognitive disorders [3-5] (HAND, a.k.a. neuroAIDS or neuroHIV, which could also progress to HIV-associated dementia, HAD).Both AD/ADRD and HAND are associated with neurotoxicity induced mainly by the disruption of neuronal Ca^2+^ homeostasis (i.e., excessive and uncontrolled increase of intracellular free Ca^2+^, [Ca^2+^]_in_), which leads to dysfunction, injury, and ultimately death of neurons, especially in the brain regions that are key regulators of neurocognitive function [6]. Furthermore, it is also worth noting that AD/ADRD is becoming more prevalent among HIV/AIDS patients during aging, even when they are on combination antiretroviral therapy (cART) [1,3].

Ironically, despite the therapeutic effects of cART on suppressing HIV-1 replication (which could ultimately protect neurons from the impact of HIV-1 even though they are not infected by the virus), antiretroviral medicines (ARVs) *per se*, which are commonly given to HIV/AIDS patients in a co-formulated regimen (a.k.a cART), are also found to be neurotoxic in the central nervous system (CNS) [7-10]. The exact mechanism(s) by which AD, HAND and the side effects of ARVs induce neurotoxicity is not fully understood; but the disturbance of neuronal Ca^2+^ homeostasis is considered as one of the critical factors. In addition, aging, and certain drugs of abuse could have further deleterious impact on AD- and HIV/AIDS-induced neurocognitive and neuropsychiatric disorders [11-15], along with the potential complications induced by cART [7-10]. The mechanism that underlies the comorbidity of these neurodegenerative conditions and aging is also unknown, and desperately needs to be elucidated.

## Background for a new hypothesis

### Dysregulation of neuronal Ca^2+^ homeostasis induces neurotoxicity in AD/ADRD and HAND:

There is rising consensus that dysregulation of neuronal Ca^2+^ homeostasis plays a crucial role in inducing dysfunction, injury, and death of neurons in the brain, which consequently disrupts neurocognitive function of AD/ADRD and HAND patients, and could ultimately drive mild cognitive impairments to more severe deficits, including dementia [6]. Previous studies have empirically attributed AD/ADRD or HIV/AIDS-induced neurotoxicity to many possible risk factors, including, but not limited to, dysregulation of Aβ and other proteins (e.g., Aβ plaques and neurofibrillary tangles composed of hyperphosphorylated tau proteins) [1,16,17], secretion of pro-inflammatory factors like chemokines and cytokines [9,18], inhibition of astrocytic glutamate (excitatory amino acid) transporters [9,19], overactivation of ionotropic glutamatergic NMDA receptors (NMDARs) [1,9,16] and voltage-gated Ca^2+^ channels (VGCCs) [1,3-5,9,16,20,21], and combinatorial effects of them. Based upon these studies, numerous plausible upstream mechanisms that may contribute to the original cause of AD/ADRD, HAND, and associated neurotoxicity are widely speculated and studied. For more than three decades, the Aβ theory is the one (though may not be the only one) dominating AD/ DRD research and treatment; and the NMDAR dysfunction, which is partially involved in inducing neuronal Ca^2+^ dysregulation and neurotoxicity in AD/ADRD and HAND, is generally considered to be a key player in the mechanism underlying AD- and HAND-induced neurotoxicity that could result in dysfunction, injury, and death of neurons.

Under these circumstances, when we ask what is the most likely mechanism that ultimately leads to the neurotoxicity found in AD and HAND, a general answer could be the disruption of neuronal Ca^2+^ homeostasis (Ca^2+^ dysregulation) and, more specifically, the excessive and uncontrollable increase of [Ca^2+^]_in_. Indeed, this downstream mechanism of Ca^2+^ dysregulation is the final common path to neurotoxicity found in the brain of AD/ADRD and HIV/AIDS patients, through which numerous disrupted upstream mechanisms converge to abnormally raise [Ca^2+^]_in_, and there by altering gene transcription and numerous cellular signaling pathways, which eventually lead to dysregulation, injury, and even death of neurons. Although the critical role of such Ca^2+^ dysregulationin inducing neurocognitive dysfunction is considered and recognized in previous studies [6], mechanisms other than the NMDAR overactivation, including, but not limited to, VGCC dysfunction, is understudied.

**Figure 1:**
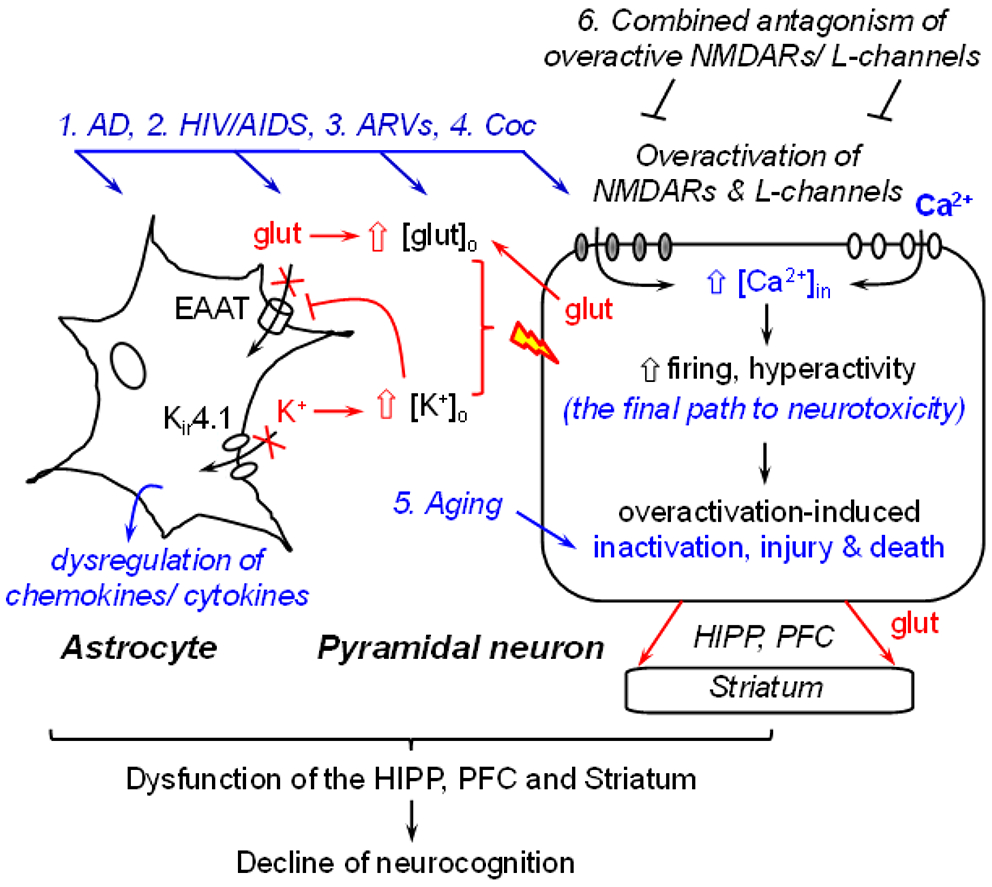
A working model for diminishing the impact of neurotoxicity induced by excessive Ca^2+^ in HIPP and mPFC pyramidal neurons that may contribute to AD/ADRD and HIV-associated dementia. AD and chronic exposure to HIV, ARVs and cocaine (1-4) are associated with abnormal increase of firing and Ca^2+^ influx *via* overactive/over-expressed NMDARs and L-type VGCCs (L-channels) among glutamatergic pyramidal neurons in the HIPP and mPFC. Such Ca^2+^ dysregulation could drive neurons from the status of overactivation to inactivation (loss of firing), injury, and eventually death. Hyperactivity of HIPP and mPFC pyramidal neurons also enhances glutamate release in the local circuit, and in their subcortical brain regions, including the striatum, which also disturbs the activity of striatal neurons. Astrocytes in the HIPP and mPFC are also altered by AD, HIV, ARVs and drugs of abuse. Besides dysregulating chemokines/cytokines and inducing inflammation, AD, HIV, ARVs and Coc also alter the functional activity of astrocytes in maintaining and regulating the extracellular glutamate ([glut]_o_) and K^+^([K^+^]_o_) homeostasis. Consequently, the ability of astrocytes to uptake glutamate via excitatory amino acid transporters, and to balance K^+^_o_ influx/efflux through K_ir_4.1 channels, are significantly reduced. These alterations result in an increased [glut]_o_ and [K^+^]_o_, which depolarize membrane potential of surrounding pyramidal neurons, and render them more susceptible and vulnerable to deleterious (e.g., Aβ, HIV-1 proteins, ARVs, and cocaine), and even physiological (e.g., glutamate) excitatory stimuli. Moreover, because glutamate uptake by astrocytes via EAAT is K^+^-dependent, increased [K^+^]_o_ also reduces EAAT’s activity. Together, the consequential effects of astrocyte dysregulation exacerbate Ca^2+^-induced neurotoxicity in pyramidal neurons. Aging also decreases the activity of pyramidal neurons (5), even without the influences of AD, HIV and Coc. This decrease likely contributes to the decline of cognition; and that could be compounded byAD-, HIV-, ARVs-, and/or Coc-induced neurotoxicity. Collectively, dysfunction of the HIPP, mPFC and striatum integratively contributes to the decline of cognition, especially during aging. It is likely that combined chronic antagonism of NMDAR/L-channel overactivation (6) will significantly diminish excessive Ca^2+^ influx via overactive NMDARs/L-channels, blocking the final path to neurotoxicity and therefore stop or at least slowdown the progression of these two neurodegenerative diseases to dementia.

The brain regions that are profoundly affected by AD [1,16,22] and HIV/AIDS [6] with associated disruption of neuronal Ca^2+^ homeostasis are key players in regulating cognition. These regions mainly include, but may not be limited to, the hippocampus (HIPP), prefrontal cortex (PFC), and dorsal/ventral striatum (a.k.a. the caudate-putamen and nucleus accumbens, respectively) [6,23]. The major type of neurons in the HIPP and PFC is glutamatergic pyramidal neurons (80~90%) [24,25]. However, these neurons are profoundly altered, functionally and structurally, by AD- and HIV/AIDS-induced Ca^2+^ dysregulation [6,23]. Such Ca^2+^ alterations in HIPP and PFC neurons also affect the functional activity of subcortical GABAergic striatal neurons [26], which receive excitatory glutamate inputs from the HIPP and PFC [6,23]. In addition, function of other types of neurons [23] and non-neuronal cells (e.g., astrocyte [17-19,27,28]) in these brain regions are also altered (either directly or indirectly) by AD or HIV-induced Ca^2+^ dysregulation (Figure 1).

It is well-known that excessive [Ca^2+^]._in_ is toxic, which could induce hyperactivity (abnormally-increased firing that can drive neurons to the status of overactivation [29]); injury (that reduces the synapses, dendritic processes, synaptic activity [30], and connectivity among neurons and glial cells [31,32]); aberrant and loss of firing (due to overactivation-induced inactivation) [29] which could eventually lead to death of pyramidal neurons in the HIPP and PFC [6,23], and decline of cognitive function, dictated by neuronal circuits. It is also worth noting that aging, *per se*, is associated with a significant decrease in the mPFC neuronal activity, which could initiate at middle age [33], even without the impact of AD or HIV/AIDS (Figure 1). Taken together, these findings strongly suggest that AD-and/or HAND-induced decrease of neuronal activity (i.e., overactivation-induced inactivation) could be worsened during aging, and such comorbid conditions will almost inevitably exacerbate the cognitive dysfunction in AD and HAND patients.

### Astrocyte dysfunction also contributes to AD- and HIV-induced neurotoxicity:

Astrocytes play a very important role in regulating the health and membrane excitability of surrounding neurons, and subsequently the neuronal activity. Both AD [18,34] and HIV [9] have a negative impact on the function of astrocytes in the HIPP and PFC. In addition to evoking the immune responses and neuroinflammation mediated by reactive astrocyte-released cytokines and chemokines, AD and HIV also alter the dynamic, functional activity of astrocytes in maintaining and regulating the extracellular glutamate and potassium (K*) homeostasis in the brain. Such astrocytic dysregulation of glutamate and K^+^results in abnormal increase in the extracellular levels of glutamate ([glut]_o_) and K^+^ ([K^+^]_o_) [17-19,27,28], two key regulators of the membrane excitability of neurons. The ability of astrocytes to uptake [glut]_o_ through glutamate transporters and to balance [K^+^]_o_ is significantly reduced in the context of AD and neuroHIV [17-19,27,28], resulting in accumulation of both [glut]_o_ and [K^+^]_o_. Such abnormally-increased [glut]_o_ and [K^+^]_o_ facilitate depolarization of the membrane potential (*V*_m_) of surrounding neurons, promoting AD and/or HIV-induced neuronal hyperactivity, which could exacerbate neurotoxicity induced by dysfunctional neurons *per se* (e.g., excessive [Ca^2+^]._in_ mediated by overactivated NMDARs and VGCCs). Eventually, such dysregulation of [glut]_o_ and [K^+^]_o_ drives hyperactive glutamatergic HIPP and PFC pyramidal neurons from overactivation to inactivation (losing firing) [13,14,29], further diminishing the role of these brain regions in regulating neurocognition (Figure 1).

Persisting high levels of [Ca^2+^]_in_ could renders glutamatergic pyramidal neurons more susceptible and vulnerable to deteriorative excitatory stimuli following Aβ aggregation [1,2,16], HIV infection [13-15,29], the side effects of ARVs (cART) [7-10,35-37], some drugs of abuse (e.g., cocaine [11,13]), and even to otherwise physiological excitatory stimuli (e.g., glutamate). Collectively, despite numerous upstream mechanisms that may underlie the neuropathogenesis of AD and HIV/AIDS, these studies reveal that (1) Excessive [Ca^2+^] _in_ in HIPP and PFC pyramidal neurons is the fundamental basis of AD- and HIV-induced neurotoxicity; (2) The function of brain regions that regulates neurocognition is significantly diminished due to excessive Ca^2+^-induced neurotoxicity among pyramidal neurons in the HIPP and PFC, as well as in striatal neurons; and (3) AD- and HIV-induced astrocyte dysfunction not only induces neuro-inflammation by dysregulating chemokines and cytokines, but also disturbs [glut]_o_ and [K^+^]_o_ homeostasis; and therefore, combinedly worsens the neurotoxicity induced by excessive [Ca^2+^]_in_ in surrounding neurons.

### Both NMDARs and VGCCs mediate AD- and neuroHIV-induced neuronal Ca^2+^ dysregulation:

NMDARs are ligand-gated ionotropic glutamatergic receptors located postsynaptically in neurons, which are highly permeable only for free Ca^2+^ when activated by binding of glutamate (or N-methyl-D-aspartate). VGCCs are transmembrane proteins located on the dendrites and cell bodies of neurons that are activated by membrane depolarization in response to excitatory stimuli, including Ca^2+^ influx through NMDARs (as well as via AMPA receptors, another subtype of ionotropic glutamatergic receptors). The NMDARs and VGCCs regulate the neuronal *synaptic* excitability and *intrinsic* excitability, respectively, owing to their localization, properties and function in synergy with other receptors, ion channels and signaling pathways to ultimately regulate neuronal activity. However, pathological conditions in both AD and HIV significantly alter the function and expression of NMDARs and VGCCs, inducing abnormally-increased influx of [Ca^2+^]._in_ in HIPP and mPFC pyramidal neurons, likely leading to exacerbated neurotoxicity.

Unfortunately, current clinical treatments approved by FDA for AD/ADRD [31,38] focus predominantly (though may not solely) on decreasing NMDAR overactivation to diminish excessive Ca^2+^ influx and consequential neurotoxicity, either with or without promoting the acetylcholine transmission. The antagonism of NMDAR overactivation (using selective NMDAR antagonists to reduce abnormally increased firing mediated NMDARs) may protect hyperactive neurons from overactivation-induced injury and death caused by excessive [Ca^2+^]._in_; while the regimen that promotes acetylcholine transmission (using inhibitors of acetylcholine metabolism that increase synaptic levels of acetylcholine) could facilitate neuronal activity (e.g., promoting firing mediated by nicotinic receptors that are activated by enhanced and prolonged effects of acetylcholine due to reduced metabolism) in the brain regions suffering from declined neuronal activity by enhancing cholinergic neurotransmission (which could increase firing mediated by nicotinic cholinergic receptors). However, given the opposite mechanism of action of these two types of medicines on neuronal activity (i.e., decreasing vs. increasing firing, respectively), it will be very difficult for a clinician to make a decision regarding whether and when these two types of medicines should be given to patients to treat AD.

So far there is no any particular drug based upon the Aβ theory that can cure AD/ADRD. This reality suggests that the current theory regarding what causes AD/ADRD and how this neurodegenerative disease progresses to dementia, needs to be re-considered. A similar condition also applies to neuroHIV research regarding treatment of HAND. A few previous clinical trial studies have tried to separately target overactivated NMDARs [39] or voltage-gated L-type Ca^2+^ channels [40] to treat HIV-associated dementia; but none of those strategies work. In addition, unlike for treating AD, there is no FDA-approved medicine to specifically treat HAND. Therefore, a novel theoretical concept associated with anew hypothesis and working model are urgently needed for future AD and HAND studies, as well as for the development of new therapeutic strategies to treat other neurodegenerative diseases that could also progress towards dementia.

### A potential novel theory:

Based upon scientific evidence and current status of drug investigations into AD and HAND, we propose a potential novel theory: overactivation of both NMDARs and VGCCs plays a key role in disturbing neuronal Ca^2+^ homeostasis and inducing pathophysiological rise of [Ca^2+^]_in_, which may be the underlying mechanism mainly responsible for the neuropathogenesis in AD and HAND. In this novel theory, the Aβ dysregulation is not considered as a causative factor of AD, but rather one of the consequential effects resulting from neuronal Ca^2+^ dysregulation [1,16]. This novel theoretical concept will likely provide an opportunity to switch the battlefield from searching the upstream mechanism(s) that may (or may not) cause AD, to blocking the downstream final common path to neurotoxicity, which could significantly diminish AD-induced injury and death of pyramidal neurons in the key brain regions (e.g., HIPP and PFC), thereby alleviating neurocognitive function. Moreover, it will also facilitate the development of new therapeutic strategies against the Ca^2+^ dysregulation-induced neurotoxicity, specifically, by concurrently antagonizing the overactivation of both NMDARs and L-channels (Figure 1). Given a similarity in neuronal Ca^2+^ dysregulation, such theoretical concept could also apply to HAND.

Meanwhile, we do acknowledge that this novel theory may not be perfect. It would still need to provide a definite and ultimate answer for what the original cause of AD (or neuroHIV) is, in order to direct future therapeutic strategies to cure AD or HAND. The exact mechanism by which AD induces overactivation of NMDARs and L-channels still needs to be defined. Although this novel theoretical concept may be expedient (until a more appropriate one occurs), normalizing the neuronal Ca^2+^ homeostasis and firing activity that are drastically altered by AD (and HAND) will very likely slowdown and diminish (if not prevent or stop) the progression, and reduce the severity, of AD/ADRD (and HAND with cART) during aging. In other words, research based upon this novel theory may bring us a more effective and practical new strategy for combating against AD and HAND. Given that the Aβ theory is almost the sole theory for AD currently, and it has not been effective in developing therapy against AD, this novel theory may actually have its potential and deserves to be investigated further.

### The novel hypothesis:

Although currently we do not have the capability to define and intervene all the plausible *upstream* mechanisms that may cause AD/ADRD or HAND, we do have the advantage and opportunity to block the *downstream* final path to neurotoxicity. Based upon the novel theory, we hypothesize that combined enduring antagonism of NMDAR and L-channel overactivation will significantly diminish excessive [Ca^2+^]_in_ and neurotoxicity, thereby reducing dysfunction, injury, and death of pyramidal neurons in the HIPP and PFC during the progression of AD and HAND; and that will improve the function of these brain regions in regulating neurocognition (Figure 1).

A great advantage of such a combined regimen is that it is feasible and does not require development of any new drug (at least for now). The medicines that can be quickly applied to block the final path to neurotoxicity include a selective NMDAR antagonist (i.e., memantine) in combination with a specific VGCC blocker (e.g., nifedipine, a blocker for L-type VGCC, L-channels). These medicines are already approved by FDA for treating AD and hypertension, respectively, for many years, and both are safe and well-tolerated by patients. This combined enduring treatment regimen will diminish AD- and/or HIV-induced neuronal Ca^2+^ dysregulation mediated by overactivated NMDARS and L-channels, thereby reducing neuronal injury and death of neurons. Ultimately, it may effectively alleviate the function of the brain regions that regulate neurocognition.

### A potential new therapeutic strategy:

To test the proposed novel hypothesis, research needs to focus on (1) Determining neuronal dysfunction in understudied brain regions that are profoundly altered by aging, AD/ADRD, or HIV/AIDS in combination with cART *in vivo*, as well as in comorbid conditions of them, (2) Identifying new drug target(s) for pharmacological intervene of neuronal Ca^2+^ dysregulation, and (3) Exploring new treatment regimen.

Current clinical and pre-clinical studies regarding AD/ADRD-induced neurocognitive impairments focus mainly on dysfunction of the HIPP, likely due to its critical role in learning and memory. However, the HIPP is not the only regulator of cognition. Other brain regions, especially the PFC and striatum, also participate in regulating neurocognition in a significant manner; while both are functionally and anatomically altered by aging [41,42], AD [2,43] and HIV/AIDS [23,44,45]. Unexpectedly, the PFC and striatum seem to receive much less attention and, therefore, are understudied compared to the HIPP. It is worth noting that hyperactivity of the PFC (with abnormally-increased [Ca^2+^]_in_) appears to be prevalent in both AD [1,16,22] and HIV/AIDS [46,47] which could induce neurotoxicity and drive overactivated pyramidal neurons to firing loss [13,29], similar to a consequence of aging [48]. Our published studies also demonstrate that, in the context of neuroHIV, hyperactivity of mPFC pyramidal neuron [13-15,29,33,48] disturbs their glutamate output to the striatum, which abnormally increases firing of GABAergic striatal neurons [26]. Thus, it is likely that similar neuronal dysregulation may also occur in AD patients. Given that (1) Decreased activity and injury/loss of HIPP and PFC neurons are associated with ADRD and HIV-associated dementia, (2) Aging likely exacerbates such conditions by inducing additional loss of neuron firing [33], and (3) ARVs-induced hyperactivity is mediated by overactivation of L-channels in mPFC pyramidal neurons following chronic treatment [49], it is the time to pay equal attention for the dysfunction of mPFC and striatum in AD and HAND, in addition to the HIPP.

As discussed above, current treatments for AD rely mainly on diminishing the impact of NMDAR overactivation. However, overactivated L-channel, another key target that also induces neuronal Ca^2+^ dysregulation and neurotoxicity, appears to be overlooked. Our recent studies demonstrate a Ca^2+^ dysregulation and neuronal hyperactivity recapitulated in HIV-1 transgenic (Tg) rats [13,29]. Importantly, we also reveal that this neuronal hyperactivity is associated with overactivation and overexpression of VGCCs (especially L-channels), either in the context of neuroHIV [13,15,29,50], or following chronic treatment of ARVs, or in the presence of Aβ (data not shown). Collectively, these studies indicate that in addition to overactivated NMDARs, dysfunctional L-channels also play a key role in inducing excessive [Ca^2+^]_in_ and neurotoxicity in mPFC pyramidal neurons [13,29]; and that must be considered in future treatment regimen.

Selecting an appropriate novel treatment regimen for treating AD and HAND is also critical. Encouragingly, the novel hypothesis is supported by outcomes from our published study [29]. This study reveals that, for the first time in the field, combined, but not individual, chronic antagonism of NMDAR and L-channel overactivation *in vivo* normalizes firing activity of hyper-excited mPFC pyramidal neurons by significantly reducing previously-increased Ca^2+^ influx via overactivated NMDARs and L-channels back to control levels in the context of neuroHIV [29]. Importantly, this study also indicates that the proposed novel hypothesis is testable and studies based upon it are feasible. Furthermore, our study also suggests that the timing for such a treatment regimen is equally crucial and important compared to the choice of medicines, considering the difficulties involved in reviving severely injured HIPP, mPFC, and striatal neurons back to physiological state from advanced stages of dementia.

## Summary and Conclusion

The failure of all current treatments and previous clinical drug trial studies based on the Aβ theory suggests that the Aβ theory in AD/ADRD may be incorrect for the interpretation of the underlying mechanism of AD/ADRD. Similar initial attempts for treating HAND have also been unsuccessful. It is likely that Aβ is not a causative factor for AD/ADRD, but one of the consequential effects of the neuronal Ca^2+^ dysregulation in this devastating neurodegenerative disease. Thus, novel theory, hypotheses, and therapeutic strategies are desperately needed for future pre-clinical studies and clinical treatments of AD/ADRD (and HAND). Although there are numerous plausible upstream mechanisms that may underlie AD/ADRD and HAND, it is biologically complicated to determine the one that originally causes AD or HAND. However, it is realistic and feasible to combat against Ca^2+^ dysregulation-induced neurotoxicity that causes dysfunction, injury, and ultimately death of HIPP and mPFC pyramidal neurons in the context of AD or HAND, specifically by concurrently and enduringly antagonizing the overactivation and/or overexpression of NMDARs and L-type VGCCs (L-channels) at early stages of their dysfunction. Such a new therapeutic strategy that locks the final path to AD and HAND-induced neurotoxicity may ultimately alleviate the neurocognitive function in AD and HIV/AIDS patients.
